# Determining the Spiritual Care Needs of Patients with Indwelling Urinary Catheterization: A Cross-Sectional Descriptive Study in Turkey

**DOI:** 10.1007/s10943-024-02010-x

**Published:** 2024-02-10

**Authors:** Yadigar ORDU, Sakine YILMAZ

**Affiliations:** 1 Faculty of Nursing, Department of Nursing, Necmettin Erbakan University, Konya, Turkey; 2 Faculty of Health Sciences, Midwifery Department, Cankiri Karatekin University, Cankiri, Turkey

**Keywords:** Urinary Catheter, Spirituality, Need for Spiritual care, Nursing

## Abstract

This study was undertaken to ascertain the spiritual care requirements of patients undergoing indwelling urinary catheterization. Identifying the spiritual care needs of patients with indwelling urinary catheterization is crucial for fortifying their resilience and fostering positive health behaviors. Furthermore, it proves pivotal in devising customized nursing interventions. A descriptive cross-sectional study involving 122 participants (50 female, 72 male) undergoing indwelling urinary catheterization within the inpatient clinics of a state hospital in Turkey was conducted. Data were meticulously gathered through a participant information form and the Spiritual Care Needs Scale. The subsequent analysis employed the Kruskal-Wallis and Mann-Whitney U tests, facilitated by the SPSS 22.0 program. This study adhered to the STROBE recommendations in reporting. The findings indicated that individuals with indwelling urinary catheters exhibit spiritual care needs exceeding the norm, with heightened scores in subdimensions such as meaning and hope, caring, and respect. A statistically significant positive association was identified, revealing a difference in spiritual care needs between women and men. Similarly, a positive association, statistically significant, was observed in the spiritual care needs of patients aged 72–98 compared to those aged 41–71. Furthermore, a positive and statistically significant difference surfaced in the spiritual care needs of patients with long-term indwelling urinary catheters as opposed to those with medium and short-term urinary catheters. Therefore, it is imperative to consider the spiritual care needs of patients undergoing indwelling urinary catheterization.

## Introduction

Urinary catheterization stands out as one of the most frequently conducted procedures in medical institutions for patients. Its application spans various scenarios, including post-surgical interventions, bladder irrigation, administration of bladder medications, resolution of urinary retention, facilitation of urine excretion, and ensuring comfort during the terminal phase of individuals (Craven et al., 2015; Perry et al., 2021). Approximately one-fourth of hospitalized patients undergo urinary catheterization, which can indicate patient frailty and disease severity. Specifically, indwelling urinary catheterization involves continuously placing a foley catheter within the bladder (Balcı Akpınar, 2014; Shimoni et al., 2022).

The impact of indwelling urinary catheterization on patients can manifest in various adverse ways. As an invasive procedure, patients undergoing indwelling urinary catheterization face an increased risk of infections. The presence of a urinary catheter emerges as a significant factor influencing in-hospital mortality among acutely hospitalized adults(Shimoni et al., 2022).

Individuals with indwelling urinary catheters often employ various methods to conceal the urinary bag during interactions with others. These efforts to conceal the catheter negatively impact both social interactions and the individual’s body image. The lack of control over urinary excretion can lead to a negative impact on self-esteem. Furthermore, the presence of an indwelling urinary catheter not only influences personal hygiene practices but also dictates clothing choices, necessitating the use of looser and more comfortable attire due to the catheter and attached urine bag (Nollen et al., 2022; Shimoni et al., 2022).

The mobility of individuals is contingent upon the presence of the urinary bag. The sexual life of individuals with indwelling urinary catheters experiences negative repercussions, primarily stemming from the deterioration of body image. Such individuals may grapple with feelings of sexual inadequacy, leading to a distancing from their partners. Additionally, the constraint imposed on movement during sleep, attributed to the presence of the urinary bag, contributes to a reduction in sleep quality (Ndomba et al., 2022; Nollen et al., 2022).

Furthermore, individuals encounter challenges in fulfilling their religious duties due to the presence of the indwelling urinary catheter (Ndomba et al., 2022). These difficulties contribute to a negative impact on individuals’ perceptions of their health status. Recognizing the importance of spirituality and spiritual care for such individuals is crucial. Spirituality emerges as a significant predictor of physical, mental, and social well-being (Wu et al., 2016). It serves as a means through which individuals comprehend themselves, safeguard their self-esteem, and navigate their problems and illnesses more easily (Hajiyousouf & Bulut, 2022).

Spiritual care stands as a subjective and dynamic concept, serving as a unique dimension that integrates with all other facets of care (Özveren et al., 2022). Its significance lies in aiding individuals in selecting healthy coping strategies, enhancing their overall life quality, and elevating the standard of care they receive. For many patients, spirituality plays a vital role in coping with illness and is integral to their quality of life. It embodies how an individual seeks meaning in their life. Moreover, spiritual needs constitute a multidimensional phenomenon influenced by religious and cultural backgrounds.

Spiritual needs encompass four primary dimensions: communication, peace, meaning/purpose, and superiority, and are influenced by underlying psychosocial, emotional, existential, and religious needs. These needs find expression through an individual’s beliefs, values, and practices. While a patient’s spiritual needs may be rooted in a specific religion, it is noteworthy that even non-religious individuals harbor spiritual needs. The meaningfulness of these needs is determined by the interplay of the patient’s attitudes and beliefs, underlying worldviews, current circumstances, and the specific cultural context. Addressing patients’ spiritual needs is a crucial component of comprehensive health care. Providing spiritual care for both patients and their families contributes to improved health outcomes, including factors such as oxygen saturation, heart rate, blood pressure, and respiratory rate, ultimately enhancing the overall quality of life. In the holistic approach to patients’ health plans, spiritual care emerges as a fundamental element. Identifying and understanding patients’ unmet spiritual needs is a prerequisite for effectively supporting and integrating these needs into their care. This recognition is essential for optimizing the overall healthcare experience (Haris et al., 2020; Rababa & Al-Sabbah, 2023; Riklikienė et al., 2020b).

Patients living with an indwelling urinary catheter commonly encounter problems such as urinary tract infections, painful bladder spasms, blockages, and leaks. Beyond these communicable complications, individuals with indwelling urinary catheters may face non-communicable challenges, including a loss of respect, job termination, erectile problems, diminished desire for sexual intercourse, and financial strain due to accumulating hospital bills. These multifaceted issues significantly contribute to the deterioration of patients’ social and psychological well-being with indwelling urinary catheters (Ndomba et al., 2022).

Patients often regard spirituality and religious rituals as pivotal elements in navigating grief, alleviating pain, and confronting critical illness. In moments of despair or helplessness, individuals frequently turn to spirituality as a source of hope, a means to alleviate anxiety and a strategy to overcome the challenges posed by grief, pain, and critical illness (Rababa & Al-Sabbah, 2023).

Patients encountering medical devices such as urinary or IV catheters, casts, or wound dressings as part of their disease management may encounter challenges in fulfilling their spiritual care needs (Haris et al., 2020). Addressing the spiritual care needs of patients undergoing urinary catheterization is particularly crucial in this scenario. The existing literature on this specific subject is limited. To the best of our knowledge, this study represents the inaugural attempt to investigate the spiritual care needs of patients undergoing indwelling urinary catheterization.

### Research Questions

The research questions are as follows:

1. What is the mean score of patients’ spiritual care needs?
2. What are the factors affecting the patients’ spiritual care needs?
3. Is there a significant difference between some descriptive characteristics of patients and their spiritual care needs?

## Materials and methods

### Study Design

The study was descriptive and cross-sectional.

### Participants

The study was conducted within the inpatient clinics of a state hospital in Turkey. The study sample comprised individuals aged 18 and above who were hospitalized in the clinics, possessed indwelling urinary catheters, exhibited no communication issues, were devoid of a neuropsychiatric disorder diagnosis, and willingly consented to participate.

The study took place from November 1, 2023, to February 19, 2023, during which an Informed Consent Form was acquired from the participating patients. The study encompassed a population of 160 patients with urinary catheters in the inpatient clinics of a state hospital. Employing the sample size calculation formula applicable to a known universe, the study determined a sample size of 120 patients with a 95% confidence interval and a 5% margin of error, as per Kılıç (2012). Ultimately, the study was conducted with a sample size of 122 patients.

### Measurements

The data were collected through the “Participant Information Form” developed by the researchers based on the relevant literature (Botlero et al., 2010; Frödin et al., 2022; Özveren et al., 2022) and “Spiritual Care Needs Scale” (Günay İsmailoğlu et al., 2019; Wu et al., 2016).

### Participant Information form

The participant information form comprises two sections encompassing a total of 15 questions. The first section comprises nine questions focused on sociodemographic data, including age, gender, marital status, and educational status. The second section incorporates six questions pertaining to aspects such as the duration of indwelling urinary catheters, knowledge of spiritual care, and engagement in religious rituals, drawing on insights from studies by Botlero et al. (2010), Frödin et al. (2022), and Özveren et al. (2022).

### Spiritual Care Needs a Scale

The Spiritual Care Needs Scale, originally formulated by Wu et al. (2016), underwent adaptation into Turkish by Günay İsmailoğlu et al. (2019), accompanied by comprehensive validity and reliability studies. The scale is structured with two sub-dimensions and encompasses a total of 21 items. The first sub-dimension, labeled “meaning and hope,” spans items 1–12 and 14, incorporating spiritual expressions directed towards nature and self. Meanwhile, the second sub-dimension, “caring and respect,” encompasses items 13 and 15–21, encapsulating spiritual expressions oriented towards other individuals.

The scale utilizes a 5-point Likert scale for scoring, with response options ranging from (1) “Not at all necessary” to (5) “Absolutely necessary.” A higher score on the scale signifies an elevated need for spiritual care among the patients. The scale’s scoring ranges from a minimum of 21 to a maximum of 105. Internal consistency, as measured by Cronbach’s Alpha, demonstrates a high level of reliability, with α = 0.93 for the entire scale, α = 0.90 for the meaning and hope sub-dimension, and α = 0.89 for the caring and respect sub-dimension, as indicated in studies conducted by Günay İsmailoğlu et al. (2019) and Wu et al. (2016).

In the current study, the Cronbach’s Alpha coefficient for the overall scale was calculated as 0.91. The specific sub-dimensions of the scale exhibited robust internal consistency, with a Cronbach’s Alpha coefficient of 0.87 for the meaning and hope sub-dimension and 0.85 for the caring and respect sub-dimension. These coefficients signify a high level of reliability and consistency in measuring spiritual care needs within the scope of the study.

### Data Collection

Data were collected by face-to-face interviews with patients with indwelling urinary catheters in inpatient clinics meeting the inclusion criteria. Each interview lasted an average of 15–20 min.

### Data Analysis

The data analysis for this study was performed using the SPSS statistical package (version 22.0; SPSS, Inc., USA). Descriptive statistics were used to summarize the data, including number, percentage, mean (minimum, maximum), median, interquartile range (IQR), and standard deviation values. The normality distribution of the data was assessed using the Kolmogorov-Smirnov test, Skewness, and Kurtosis. In cases where the data did not follow a normal distribution, the Kruskal-Wallis and Mann-Whitney U tests were applied for statistical comparisons.

## Results

The patients in this study had a mean age of 72.17 ± 12.28 years, with 55.7% falling within the 72–98 age range. Regarding gender distribution, 59% were male, and the majority (68.9%) were married. Educational backgrounds varied, with 63.1% having completed primary school. A significant proportion of patients (94.3%) were unemployed, and 57.4% were retired. Financially, 52.5% reported an income equal to their expenditure. Health-related characteristics indicated that 76.2% of the patients had chronic diseases and were on continuous medication (Table 1).

**Table 1:** Distribution of patients based on some descriptive characteristics (*n* = 122)

Characteristics	n	%
*Age (year) (x̄±SD = 72.17 ± 12.28)*		
41–71	54	44.3
72–98	68	55.7
*Gender*		
Female	50	41.0
Male	72	59.0
*Marital status*		
Married	84	68.9
Single	38	31.1
*Education status*		
Illiterate	30	24.6
Primary School	77	63.1
Middle School	5	4.1
High School	6	4.9
University	4	3.3
*Employment status*		
Working	7	5.7
Not working	115	94.3
*Profession*		
Officer	4	3.3
Tradesmen	4	3.3
Retired	70	57.4
Housewife	44	36.1
*Income status*		
Income less than expenditure	45	36.9
Income equal to expenditure	64	52.5
Income more than expenditure	13	10.7
*Chronic disease*		
Yes	93	76.2
No.	29	23.8
*Continuous use of medication*		
Yes	93	76.2
No.	29	23.8

Note. Abbreviation: x̄ = mean; SD = Standard deviation

The study revealed that 68.9% of the patients had a history of prior urinary catheterization, and the majority (63.1%) currently had a short-term indwelling urinary catheter. Additionally, 59.8% of patients had their perineum cleaned by a caregiver. Regarding awareness, 63.1% of the participants were unfamiliar with the concept of spiritual care. However, 65.6% reported regularly engaging in religious rituals, while 42.6% described their understanding of spirituality at a moderate level (refer to Table 2 for detailed information).

**Table 2:** Some characteristics of patients regarding urinary catheter and spiritual care (*n* = 122)

Characteristics	n	%
*Previous urinary catheter insertion*		
Yes.	84	68.9
No.	29	23.8
I do not remember	9	7.4
*Duration of indwelling urinary catheter*		
Short term	77	63.1
Medium term	33	27.0
Long-term	12	9.8
Perineal cleansing		
Self.	49	40.2
Caregiver	73	59.8
*Knowing the concept of spiritual care*		
Yes.	45	36.9
No.	77	63.1
*Performing religious rituals regularly*		
Yes.	80	65.6
No.	42	34.4
*Level of defining spirituality*		
Bad level	30	24.6
Medium level	52	42.6
Good level	40	32.8

Note. Abbreviation: x̄ = mean; SD = Standard deviation

The analysis indicated that the median total score on the spiritual care needs scale was 78.0, with an interquartile range (IQR) of 34.0. Further breakdown revealed a median score of 47.0 (IQR = 24.0) for the meaning and hope subscale and a median score of 31.0 (IQR = 14.0) for the caring and respect subscale (Table 3).

**Table 3:** Mean Scores of the Spiritual Care Needs Scale and Its Subscales (*n* = 122)

Scale	Median (IQR)	Min	Max
Meaning and hope	47.0 (24)	13.00	65.00
Care and respect	31.0 (14)	8.00	40.00
Spiritual care needs scale total score	78.0 (34)	21.00	105.00

Note. Abbreviation: IQR = Interquartile range

Statistical analysis revealed significant differences between age groups in the median total score of spiritual care needs and the median scores of both the meaning and hope and caring and respect sub-dimensions. Specifically, patients aged 72–98 exhibited statistically significantly higher scores than those aged 41–71 (*p* < 0.05). Furthermore, a gender-based difference was observed, with the median total score of spiritual care needs and the median scores of the meaning and hope sub-dimensions being statistically significantly higher in female patients compared to male patients (*p* < 0.05). Additionally, patients with long-term indwelling urinary catheters had significantly higher spiritual care needs scores compared to those with medium and short-term catheters.

The statistical analysis revealed a significant difference (*p* < 0.05) in the median total score of spiritual care needs, meaning and hope, caring, and respect sub-dimensions among patients whose perineal hygiene was performed by a caregiver compared to those who performed perineal hygiene themselves. Additionally, a statistically significant difference was observed in the median total score of spiritual care needs, meaning and hope, caring, and respect sub-dimensions between patients who regularly performed religious rituals and those who did not engage in religious rituals regularly (*p* < 0.05).

Upon investigating the source of the observed differences, it was identified that the discrepancy was attributed to the group with long-term indwelling urinary catheters. Specifically, the median total score of spiritual care needs and the meaning and hope sub-dimension scores were higher and statistically significant in individuals with long-term indwelling urinary catheters^C^ compared to those with medium^B^ and short-term catheters (*p* < 0.05).

A statistically significant difference (*p* < 0.05) was identified in the median total score of spiritual care needs, as well as the sub-dimensions of meaning and hope, caring, and respect among patients who defined spirituality at good and moderate levels. Subsequent post-hoc comparisons revealed that all groups exhibited significant differences from each other concerning their levels of defining spirituality.

The analysis revealed that the median total score for spiritual care needs, as well as the sub-dimensions of meaning and hope, caring, and respect, were significantly higher in individuals defining spirituality at a good level^C^ compared to those defining it at a moderate level^B^. Furthermore, those defining spirituality at a moderate level^B^ had significantly higher scores compared to those defining it at a bad level (A) (*p* < 0.05) (see Table 4 for detailed information).

**Table 4:** Comparison of the mean scores of the Spiritual Care Needs Scale and its subscales with some descriptive characteristics (*n* = 122)

Characteristics	Spiritual care needs scale total mean score	Mean scores of the subscales of the spiritual care needs scale	
		Meaning and hope	Caring and respect
	Median (IQR)	Median (IQR)	Median (IQR)
*Age*			
41–71	68.5 (42.75)	41.5 (29.0)	27.5 (20.25)
72–98	85.5 (29.0)	54.0 (18.0)	33.0 (9.75)
Test statistics	Z= -3.960	Z= -4.041	Z= -3.365
	*p* = 0.001	*p* = 0.001	*p* = 0.001
*Gender*			
Female	86.5 (40.0)	55.0 (29.0)	33.0 (14.25)
Male	74.0 (30.25)	44.0 (20.75)	30.0 (13.0)
Test statistics	Z= -2.257	Z= -2.388	Z= -1.759
	*p* = 0.024	*p* = 0.017	*p* = 0.079
*Duration of indwelling urinary catheter*			
Short term^A^	75.0 (30.5)	46.0 (22.0)	30.0 (13.5)
Medium term^B^	74.0 (38.5)	45.0 (25.5)	29.0 (14.5)
Long-term^C^	96.0 (28.75)	60.0 (23.0)	35.5 (7.5)
Intergroup differences	A,B < C	A,B < C	
Test statistics	KW = 6.625	KW = 7.052	KW = 4.448
	*p* = 0.036	*p* = 0.029	*p* = 0.108
*Perineal cleaning*			
Self	67.0 (43.5)	41.0 (28.0)	25.0 (19.5)
Caregiver	86.0 (28.0)	53.0 (18.0)	34.0 (10.0)
Test statistics	Z= -4.362	Z= -4.285	Z= -3.982
	*p* = 0.001	*p* = 0.001	*p* = 0.001
*Performing religious rituals regularly*			
Yes	88.0 (22.5)	55.0 (16.75)	34.0 (7.75)
No	61.0 (37.0)	34.0 (26.5)	22.0 (16.5)
Test statistics	Z= -5.905	Z= -5.729	Z= -5.509
	*p* = 0.001	*p* = 0.001	*p* = 0.001
*Level of spirituality definition*			
Bad level^A^	39.0 (37.0)	21.5 (19.25)	16.0 (18.5)
Medium level^B^	74.0 (20.0)	46.5 (11.75)	30.0 (10.0)
Good level^C^	95.5 (12.5)	60.0 (8.75)	36.0 (4.75)
Intergroup differences	A < B < C	A < B < C	A < B < C
Test statistics	KW = 63.589	KW = 63.614	KW = 52.356
	*p* = 0.001	*p* = 0.001	*p* = 0.001

Note. Abbreviation: IQR = Interquartile range. KW = Kruskal Wallis Test; Z = Mann-Whitney U test; p = Significant value

## Discussion

Indwelling urinary catheters have been recognized as influencing various aspects of patients’ quality of life, spanning biological, physical, psychological, sociocultural, environmental, and spiritual dimensions (Fumincelli et al., 2017). The significance of spiritual care in enhancing the quality of life has been emphasized in prior research (Ndomba et al., 2022; Rababa & Al-Sabbah, 2023). Consequently, understanding the spiritual care needs of patients and identifying factors impacting their well-being is crucial. Remarkably, no existing literature has explored the spiritual needs of patients, specifically with indwelling urinary catheters. This pioneering study fills this gap, offering valuable insights for future research.

The outcomes of our study revealed that patients with indwelling urinary catheters scored an average of 78.0 (out of a maximum of 105 points) on the spiritual care scale. This finding suggests that the spiritual care needs of these patients surpass a moderate level, indicating a heightened requirement for additional spiritual care. When compared to studies examining the spiritual care needs of diverse sample groups, it is noteworthy that the spiritual well-being of cancer patients typically falls within the upper-middle-range (Riklikienė et al., 2020; Riklikienė, Tomkevičiūtė, Riklikienė et al., 2020a, b). Conversely, individuals with mobility disorders have encountered challenges in fulfilling their spiritual care needs (Bakar et al., 2014).

A study conducted in 2020 reported low spiritual care needs among cancer patients who were not in the terminal stage (Riklikienė et al., 2020a, b). Considering the unique circumstances of the patients in our study, where 63.1% were unfamiliar with the concept of spiritual care, they were in a hospital setting, and the presence of an indwelling urinary catheter impacted their quality of life significantly, there is a possibility that their spiritual care needs were indeed above a medium level. Moreover, the fact that 65.6% of the patients regularly performed their religious rituals is notable. However, the indwelling urinary catheter posed a challenge, hindering them from fulfilling the obligatory five daily prayers in the Islamic religion. This limitation may have influenced the patients’ heightened need for spiritual care.

In line with the findings of this study, patients scored an average of 47.0 (out of a maximum of 65 points) on the meaning and hope sub-dimension and 31.0 (out of a maximum of 40 points) on the caring and respect sub-dimension. Notably, in both sub-dimensions, patients scored above the middle level, indicating a notable presence of spiritual care needs. A study conducted in Taiwan yielded similar results, indicating that patients with diverse religious beliefs attained high scores in the meaning and hope, caring, and respect sub-dimensions (Wu et al., 2016).

Examining the patients in the sample group of this study, it can be inferred that individuals with indwelling urinary catheters may contend with complications such as obstruction, leakage, diminished respect, challenges in sexual intimacy, and financial burdens resulting from hospitalization. These factors significantly impact patients’ social and psychological well-being with indwelling urinary catheters (Ndomba et al., 2022). In times of despair or helplessness, individuals frequently turn to spirituality, seeking solace from a higher power to foster hope, alleviate anxiety, and navigate through grief, pain, and critical illness (Rababa & Al-Sabbah, 2023).

Muslim patients often regard prayer as an integral component of spiritual care, viewing it as essential for survival and a means to maintain proximity to Allah, seek assistance, and nurture hope (Ismail et al., 2018). In the context of this study, where the sampled patients were Muslims and likely turned to Allah in coping with the challenges posed by the indwelling urinary catheter, it is plausible that their reliance on spiritual practices influenced both sub-dimension scores of the scale to surpass the middle level.

The study uncovered a noteworthy finding that the spiritual care needs of patients with long-term indwelling urinary catheters were significantly higher. Challenges in meeting spiritual care needs have been identified concerning various medical tools, such as urinary or IV catheters, casts, or wound dressings, commonly applied in hospital settings (Haris et al., 2020). In conservative Islamic societies, adhering to religious and cultural norms, spiritual care is often fulfilled through practices like prayer and listening to the Qur’an. Maintaining cleanliness is emphasized during these practices, adding to the complexity of meeting spiritual needs in the context of medical interventions (Rababa & Al-Sabbah, 2023).

Aligning with a previous study’s findings, it was established that patients experienced unmet spiritual care needs, often stemming from feeling unclean due to medical devices like urinary or IV catheters, casts, or wound dressings. The inability to perform ablution for prayer further compounded the challenge (Haris et al., 2020). The parallels between the challenges faced by the patients in this study, where 65.6% regularly performed religious rituals, and the extended duration of indwelling urinary catheters suggest a potential correlation with heightened spiritual care needs.

This study revealed that patients in the 72–98 age range exhibited higher spiritual care needs. Consistent with this finding, previous studies by Riklikienė et al. (2020a, b)d ssing et al. (2015) have reported that individuals aged 70 and above tend to have higher spiritual needs compared to those aged 70 and under. Interestingly, there is variability in findings across studies, as Wu et al. (2016) observed a higher need for spiritual care in younger patients in a different investigation. Notably, the effectiveness of religious practices and the strength of religiosity are reported to increase with age, especially as individuals approach the end of life (Riklikienė et al., 2020a, b).

The elevated spiritual care needs observed in patients aged 72–98 in this study could potentially be influenced by several factors. As individuals approach the end of life, there tends to be an increase in the strength of faith and a heightened awareness of the proximity to death. Additionally, the accumulation of chronic diseases and increased restrictions associated with advancing age may contribute to an elevated level of spiritual care needs. The intersection of these factors may underscore the significance of spiritual well-being in the older age group, highlighting the importance of addressing spiritual care needs in the context of healthcare for this demographic.

The study revealed that women had higher spiritual care needs compared to men. This finding aligns with existing literature, where studies, such as those by Büssing et al. (2015)d cker et al. (2014), consistently reported that women exhibit higher spiritual care needs than men. The observed gender disparity in spiritual care needs may be influenced by the fact that women are generally more willing to openly express their emotions compared to men, as noted in studies such as Wu et al. (2016). This inclination toward emotional expression may contribute to the variation in the need for spiritual care based on gender observed in the current study.

The study uncovered a noteworthy correlation, indicating that patients whose perineal cleaning was performed by a caregiver exhibited higher spiritual care needs. This finding is particularly significant as a dearth of existing literature explores this specific relationship. It underscores the importance of patient relatives and nurses in playing a crucial role in providing spiritual care to patients, as highlighted in studies like Paal et al. (2015). The nuanced connection between caregiving activities, such as perineal cleaning and heightened spiritual care, merits further exploration to better understand the dynamics at play in patient well-being.

Indeed, the act of having one’s perineum cleaned by someone else may evoke feelings of embarrassment and a sense of inadequacy in self-care. The notable increase in caregiver dependency observed in this study could be attributed to the advanced mean age of the patients (72.17 ± 12.28) and the prevalence of chronic diseases among them (76.2%). In Turkish society, where caregivers for the elderly are predominantly female family members (Kırışık & Yaylagül, 2020), the likelihood of embarrassment and spiritual care needs may be further influenced. Given that 59% of the patients in this study were males and the caregivers were predominantly women, this gender dynamic, along with cultural considerations, might have contributed to the emergence of spiritual care needs tied to feelings of embarrassment.

The study revealed that patients who regularly performed religious rituals exhibited higher spiritual care needs. This aligns with findings from a recent study indicating that patients who identified themselves as religious were more likely to have spiritual care needs (Riklikienė et al., 2020). Additionally, a separate study identified that Christians who consistently engaged in religious activities had significantly higher scores in both subdimensions of spiritual care (Wu et al., 2016). These consistent findings emphasize the intricate interplay between religious practices and heightened spiritual care needs among patients, underscoring the importance of recognizing and addressing these needs in healthcare settings.

The need for spiritual care can indeed vary based on religious affiliations, sociodemographic characteristics, and cultural factors (Wu et al., 2016). Notably, a study by Mat-Nor et al. (2019) highlighted that spiritual care and recitation of the Holy Quran are effective, safe, accessible, and cost-effective non-pharmacological interventions for Muslim patients to reduce stress and anxiety. Conducting the present study with Muslim patients in Turkey introduces a specific cultural and religious context, potentially influencing the perception and priority of spiritual care needs based on the order of religious rituals performed. This underscores the importance of considering such contextual factors when addressing and tailoring spiritual care interventions for diverse patient populations.

The study identified that patients who defined spirituality at a good level exhibited significantly higher spiritual care needs. This finding resonates with a previous study where patients reported unmet spiritual problems, citing feelings of being unclean due to medical tools (Haris et al., 2020). Similarly, in the present study, the inability of patients to fulfill their spiritual care needs due to indwelling urinary catheters might have contributed to the heightened spiritual care needs among those who defined spirituality at a good level. Additionally, the substantial percentage (65.6%) of patients regularly performing religious rituals could further contribute to this result, emphasizing the interconnectedness of religious practices and spiritual care needs in the context of healthcare.

### Limitations

There are some limitations to this study. It was restricted to patients with indwelling urinary catheters who were treated in the inpatient clinics of a state hospital in Turkey and agreed to participate in the study. It is, therefore, impossible to generalize this study’s results to the entire population. In addition, it is a cross-sectional descriptive study, so causal relationships between variables cannot be established. The limitations of the study are that it is a “single-center study” and “consists only of hospitalized patients where the study was conducted.” Additionally, this study has a small “convenience” sample that may or may not characterize patients with indwelling urinary catheters in Turkey more generally.

## Conclusions

This study discerned that patients with indwelling urinary catheters exhibited spiritual care needs, and the sub-dimension scores of meaning and hope, caring, and respect were above average. Notably, a statistically significant difference was identified in the spiritual care needs of women. Furthermore, significance was observed in the spiritual care needs of patients aged 72–98 compared to those aged 41–71. Additionally, a significant difference was noted in the spiritual care needs of patients with long-term indwelling urinary catheters in comparison to those with medium and short-term catheters.

Significant observations emerged in the study regarding spiritual care needs. Patients who had their perineal hygiene attended to by a caregiver exhibited significantly higher spiritual care needs compared to those who performed perineal hygiene themselves. Similarly, patients regularly engaging in religious rituals demonstrated significantly elevated spiritual care needs compared to those who did not. Furthermore, there was a noteworthy finding that individuals defining their spirituality at a good level had greater spiritual care needs than those defining it at a moderate level, and those defining it at a moderate level had higher spiritual care needs than those defining it at a bad level.

Based on the study findings, it is recommended that nurses actively assess and inquire about the spiritual care needs of patients when providing care for individuals with indwelling urinary catheters.
